# Prediction of Biological Functions on Glycosylation Site Migrations in Human Influenza H1N1 Viruses

**DOI:** 10.1371/journal.pone.0032119

**Published:** 2012-02-15

**Authors:** Shisheng Sun, Qinzhe Wang, Fei Zhao, Wentian Chen, Zheng Li

**Affiliations:** Laboratory of Functional Glycomics, College of Life Sciences, Northwest University, Xi'an, People's Republic of China; Institut Pasteur, France

## Abstract

Protein glycosylation alteration is typically employed by various viruses for escaping immune pressures from their hosts. Our previous work had shown that not only the increase of glycosylation sites (glycosites) numbers, but also glycosite migration might be involved in the evolution of human seasonal influenza H1N1 viruses. More importantly, glycosite migration was likely a more effectively alteration way for the host adaption of human influenza H1N1 viruses. In this study, we provided more bioinformatics and statistic evidences for further predicting the significant biological functions of glycosite migration in the host adaptation of human influenza H1N1 viruses, by employing homology modeling and *in silico* protein glycosylation of representative HA and NA proteins as well as amino acid variability analysis at antigenic sites of HA and NA. The results showed that glycosite migrations in human influenza viruses have at least five possible functions: to more effectively mask the antigenic sites, to more effectively protect the enzymatic cleavage sites of neuraminidase (NA), to stabilize the polymeric structures, to regulate the receptor binding and catalytic activities and to balance the binding activity of hemagglutinin (HA) with the release activity of NA. The information here can provide some constructive suggestions for the function research related to protein glycosylation of influenza viruses, although these predictions still need to be supported by experimental data.

## Introduction

Influenza virus can cause occasional pandemics and seasonal epidemics in humans [1]. At the beginning of an influenza pandemic, preexisting immunity to the newly emerging virus is generally low in humans; thus, the virus can easily transfer from one person to another and rapidly spread across the globe. Later, on the one hand, immune antibodies to the virus are gradually induced in the host, decreasing the virulence and transmissibility of the virus. While on the other hand, the pandemic virus undergoes gradual changes in its antigenic structure (called antigenic drift) so as to escape the immune pressure imposed by the host. Such pressure and drift lead to the transformation of the pandemic virus to a seasonal one as well as the subsequent evolution of the seasonal influenza virus [1], [2], [3], [4].

Protein glycosylation is believed to be involved in the evolution of influenza viruses [5], [6]. Variation in protein glycosylation is a more efficient mechanism than even the direct mutation of amino acids for the virus to escape the surveillance of the host immune system because the glycans themselves are host-derived and hence considered as “self” by the immune system [7]. The HA and NA glycosylation of an influenza strain can affect its host specificity, virulence and infectivity either directly, by changing the biologic properties of HA and NA [8], or indirectly, by attenuating receptor binding [9], [10], [11], [12], [13], masking antigenic regions of the protein [14], [15], [16], impeding the activation of the protein precursor HA0 via its cleavage into the disulfide-linked subunits HA1 and HA2 [17], [18], [19], regulating catalytic activity or preventing proteolytic cleavage of the stalk of NA [20], [21], [22].

Previous reports showed that the seasonal H1N1 viruses possessed more N-glycosylation sequons in their HA sequences than the 1918 H1N1 strain (A/South Carolina/1/18) and it played an important roles in the host adaptation of the viruses [5], [6], [23]. Using a sequence-driven approach, Zhang *et al.* [6] found that the number of potential glycosylation sites (glycosites) in human H1N1 viruses oscillates between five and nine sites. Using a similar genome-based method, Wei *et al.* [5] indicated that there were more glycosites on the head of HA in human seasonal influenza H1N1 viruses than that in human pandemic H1N1 viruses. In addition, they proved that two highly conserved glycosites (Asn 142 and Asn 177) acquired in the RBD-A region of HA in the seasonal strains (represented by A/New Caledonia/20/1999) endow the seasonal virus with resistance to antibodies directed against both of the pandemic strains from 1918 and 2009. Das *et al.* [24] reported that the number of glycosylation sites in the HA globular domain could focus sequence variation on those regions unshielded by glycosylation. Wu *et al.* [22] also showed that the distinct N-glycan profiles of NA from the 1918 pandemic influenza virus might cause viral resistance to proteinase digestion as well as high infectivity. In our previous work, by using a series of bioinformatics tools, we found that increase of glycosite numbers was mainly occurred in the early evolutionary stages of human seasonal influenza A/H1N1 viruses, while glycosite migration (location alteration of glycosites) became the dominating mode in the later evolutionary stages. Importantly, we elucidated that the positional conversion of glycosites might be a more effective mode of glycosite alteration for the evolution of influenza A/H1N1 viruses, by analyzing the speed of a new mutant strain overtakes its original one [25].

In this study, we provided more bioinformatics and statistic data to further predict the significant biological functions of glycosite migration in the host adaption of human influenza H1N1 viruses. Several possible biological functions of glycosite migration in human H1N1 viruses were summarized in this paper. These predictions still needs to be supported by experimental data, the information here can provide some constructive suggestions for the research related to the functions of protein glycosylation in influenza viruses.

## Materials and Methods

### Protein sequence data and 3D structure of HA and NA from influenza A/H1N1 viruses

Full-length amino acid sequences of HA and NA from human seasonal influenza A (H1N1) viruses were downloaded from the influenza virus resource at the national center for biotechnology information (NCBI) (http://www.ncbi.nlm.nih.gov/genomes/FLU) [26], [27] as of March 30, 2010. The crystal structure of A/puerto rico/8/1934 HA (PDB code: 1RU7), A/California/04/2009 HA (PDB code: 3LZG) and an influenza A (H5N1) NA (PDB code: 2hty) were downloaded from PDB database (http://www.rcsb.org).

### Prediction of potential N-glycosylation sites and statistic analysis of amino acid variability

Sequon Finder was used to predict N-glycosylation sites on HA and NA. Sequon Finder is a custom-made program that just simply finds all sequons (N-X-S/T, where X is not P) within protein sequences and supposes all of sequons as potential glycosylation sites [25]. The locations of the glycosylation sites on HA and NA were numbered according to the full- length HA sequence of South Carolina/1/1918 and the full-length NA sequence of Brevig Mission/1/1918, respectively. The program is available upon request.

Amino acid variability at each position of HA and NA was quantified by counting the number of different amino acids found at the position. If a position where all sequences in a group have the same amino acid, the value of variability is set as 0. While for example a variability value of 3 corresponds to a position that has 4 different possible amino acids. The amino acid variability at each antigenic site was quantified by summing up the numbers of variability at all locations in this antigenic site. The conversation of tryptic cleavage sites in the stalk region of NA at each time period were obtained by calculating the percentage of Lysine and Arginine appeared at each site.

### Homology modeling, *in silico* protein glycosylation and visualization

The 3D structures of representative HA and NA proteins with different patterns of potential N-glycosites in human seasonal influenza A (H1N1) viruses were generated using SWISS-MODEL (http://swissmodel.expasy.org/) [28]. The crystal structure of A/puerto rico/8/1934 HA (1RU7) and A/California/04/2009 HA (3LZG) were used as the HA models of the human influenza H1N1 viruses before and after 2000, respectively. An influenza A (H5N1) NA (2hty) was used as the NA model. After homology modeling, glycans were added onto the potential N-glycosites of HA and NA using the Glyprot Server (http://www.glycosciences.de/modeling/glyprot/) [29]. Complex glycan structures were selected for all accessible sites, and the terminal sialic acid residues were manually removed in order to model the natural state of the viral glycans. All of the figures were generated and rendered using MacPyMOL [30].

## Results

### Glycosite migrations on the top of the HA head

**Figure 1:**
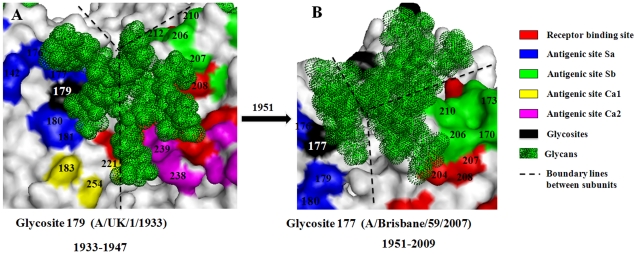
Structural overviews of the glycans attached to glycosites 179 (A) and 177(B) and their shielding regions on HA of human seasonal influenza H1N1 viruses. The glycosites are numbered in white.

**Figure 2:**
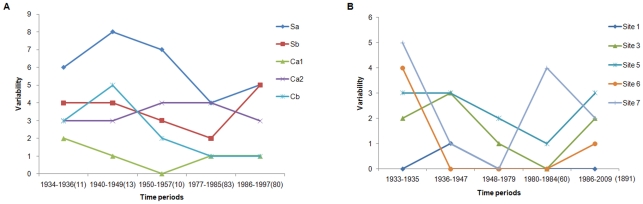
Amino acid variability at antigenic sites of HA (A) and NA (B) in human seasonal influenza H1N1 viruses. The variability was the total number of amino acids present at each antigenic region in the group of isolates with specific glycosite pattern. The numbers of corresponding strains in each group used for the analysis are given in the brackets.

Glycosite 179, appearing on the top of the HA head in human influenza H1N1 viruses in 1933, was replaced by glycosite 177 in 1951 (Figure S1) [25]. Our modeling results indicated that the glycans on glycosites 179 and 177 may not only shield the Sa site of the same subunit (since both glycosites locate at the Sa site), but also part of the antigenic sites Ca2 and Sb on the adjacent subunit as well, respectively (Figure 1). However, glycosite 179 may also obstruct the binding between the receptor binding site of the adjacent subunit and the host receptor (Figure 1a). This may be one of the reasons that glycosite 179 was replaced by glycosite 177 in 1951. The variability analysis on antigenic sites of HA also showed that the amino acid variations decreased at the antigenic site Sb but increased at the antigenic site Ca2 after 1951, which supported the modeling results to a certain degree (Figure 2a).

**Figure 3:**
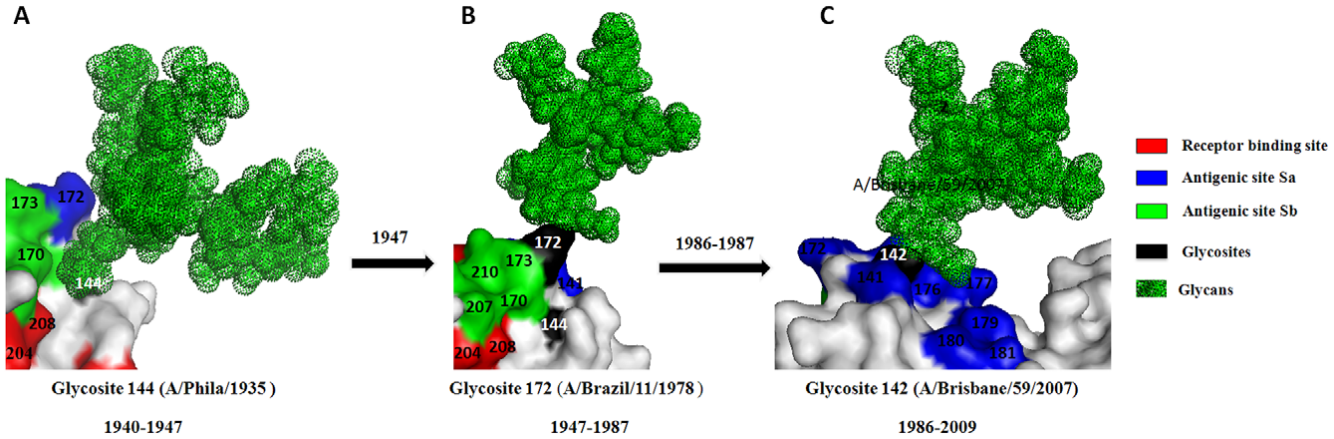
Structural overviews of the glycans attached to glycosites 144 (A), 172 (B) and 142 (C) and their shielding regions on HA of human seasonal influenza H1N1 viruses. The glycosites are numbered in white.

Glycosite 144 appeared on the top of the HA head in human influenza H1N1 viruses in 1940 and was replaced by glycosite 172 in 1947 (Figure S1) [25]. Then, the acquisition of glycosite 142 in 1986 may have rendered glycosite 172 unnecessary because glycosite 172 ultimately disappeared in 1987. The glycans at glycosites 142 may shield the antigenic site Sa more effectively because it is located at the center Sa, while glycosites 172 is at the edge of the antigenic site and glycosite 144 is adjacent to Sa (Figure 3). That should also be one of the important reasons why the amino acid variations of HA at Sa site after 1940 continuously decreased till 1985(Figure 2a).

The glycosite migrations between different regions may collaborate with each other. For example, the glycans at glycosite 144 may be better in shielding antigenic site Sb than glycans at glycosites 172 and 142, but since glycans at glycosite 177 on the adjacent subunit can shield this antigenic site well, the glycosite 142 become more preponderant than glycosite 144 as it is better in protecting antigenic Sa site. In fact, these glycosite migrations may result in totally different antigenic activity for influenza H1N1 viruses. Previous reports had shown that there was no cross-protection existed between H1 vaccines produced before and after 1986 (Table S1) [31]. Our analysis revealed that this might be due to the glycosites migrations from site 172 (and/or 144) to site 142 and/or from sites 286 and 104 to site 71, because all three vaccine strains before 1986 had the same glycosite patterns (without glycosites 71 and 142) on HA, but glycosites 172 (and/or 144) and 286 (and/or 104) had been replaced by glycosites 142 and 71 since 1986, respectively (Table S2). Besides, glycosite 365 was also replaced by glycosite 434 in 1986 which might also have some effects on the cross-protection of vaccines (see below and Table S3).

### Glycosite migrations on the side of the HA head

**Figure 4:**
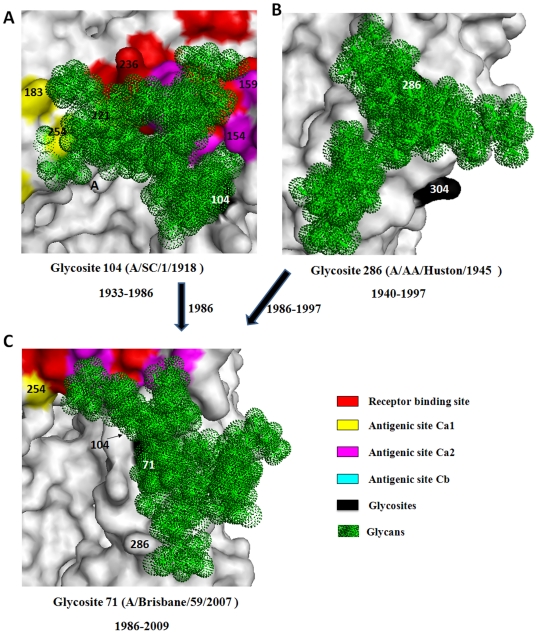
Structural overviews of the glycans attached to glycosites 104 (A), 286 (B) and 71 (C) and their shielding regions on HA of human seasonal influenza H1N1 viruses. The glycosites are numbered in white.

Region B belongs to the vestigial esterase domain which may have played a role as a fusion protein that inserted the virus into an ancestral membrane before giving rise to the modern version of HA [32]. In this region, glycosites 286 and 104 had existed since 1933 and 1940, respectively (Figure S1) [25]. The glycans on glycosite 104 can effectively shield Ca2 and part of the antigenic site Ca1 on the adjacent subunit, but it may also shield part of the receptor binding site (Figure 4a). The glycans on glycosite 286 may predominately shield a region below this site (Figure 4b). However, the glycans on glycosite 71 can shield glycosites 104 and 286 as well as part of Ca2 (Figure 4c). Thus, glycosite 71 might be highly advantageous for the prevalence of influenza viruses in humans thereby making glycosylation at glycosites 104 and 286 unnecessary. Besides, we speculated that the glycosite migrations in this region may also have some positive effects on the membrane fusion activity of human seasonal influenza H1N1 virus.

### Glycosite migrations on the head of NA

**Figure 5:**
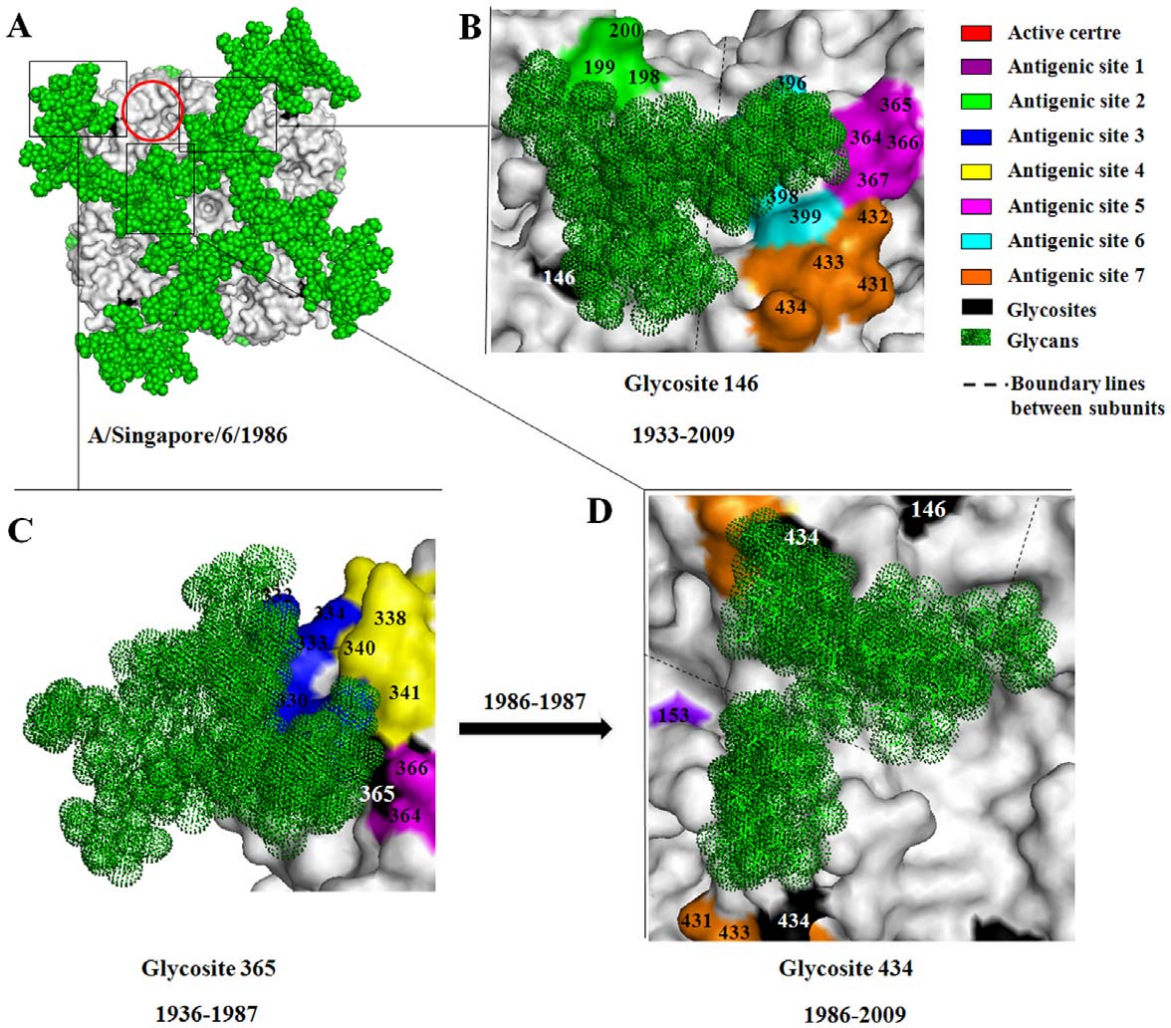
Structural overviews of the glycosylated tetramer from A/Singapore/6/1986 (A) and the shielding regions of the glycans attached to glycosites in region C, including glycosites 146 (B), 365 (C) and 434 (D). The glycosites are numbered in white.

Among four glycosites (glycosites 146, 365, 434 and 455) on the head of each NA monomer in human seasonal influenza H1N1 viruses, three of them (except glycosite 455) were located around the enzymatic active site (Figure 5a). The glycan attached to glycosite 146 could effectively shield antigenic site 1 and antigenic site 6 of the neighboring subunit of the tetrameric NA (Figure 5b). This shielding might be necessary for the function of NA and for the survival of the virus because glycosite 146 was highly conserved in almost all strains regardless of the host, and it might also be one of the important reasons that why the amino acid variations at antigenic sites 1 and 6 were very low in the evolution of human influenza viruses (Figure 2b). Glycosite 365, appearing on the head of NA in 1936, was replaced by glycosite 434 in 1986–1987 (Figure S1) [25]. Glycosites 365 and 434 are located at antigenic sites 5 and 7, respectively. The glycan attached to glycosite 365 could shield antigenic sites 3 and 5 (Figure 5c), while the glycan attached to glycosite 434 could only shield part of antigenic site 7 (Figure 5d). But glycosite 434, like glycosites 146 and 455, is at the subunit interface. The glycans at these glycosites could effectively shield the domain that joins two subunits and thus may stabilize the NA tetramer (Figure 5a). Therefore, the stability of the NA tetramer may also be very important, perhaps occasionally even more important than the resistance of NA to antibodies, to the prevalence of the virus in humans. That may be why glycosite 365 on NA was replaced by glycosite 434 in 1986–1987.

The amino acid variability analysis showed that after glycans were located at glycosite 365 (1936–1984), the amino acid variation at antigenic sites 3 and 5 continually decreased (Figure 2b). Although the amino acid variation at antigenic site 7 also decreased during 1933–1979 (Figure 2b), it dramatically increased in the time periods of 1981–1984, which might mean that the immune pressures imposed at this site by host were rising. The glycosite migration to this antigenic site (glycosite 434 on NA) might just to meet the need of resistance against these immune pressures for influenza H1N1 viruses.

### Glycosite migrations in the stalk regions of NA

Two glycosite migration (site 50 to 44 in 1947, and site 68 to 79 in 1980) occurred in the NA stalk region of the human seasonal influenza H1N1 viruses [25]. The stalk region of NA is probably the most exposed and vulnerable region to protease attacks, as NA is frequently released from viral particles through proteolytic cleavage of this region [22], [33], [34], [35]. Our research showed that the NA of the seasonal influenza viruses generally had two or three tryptic cleavage sites, but there was none existed in 1918 viruses and only one (which is also different from those in seasonal viruses) existed in pandemic 2009 viruses(Table S4). The glycans at all glycosites in the NA stalk region as well as at glycosite 235 on the head of NA (this site is near the enzymatic cleavage site between the stalk and the head of NA. Data was not shown) might be involved in the protection of viral NA against the host proteases. However, the glycans at glycosites 44 and 70 may be more effective than glycosites 50 and 68 in protecting the NA stalk against the host proteases, and thus glycosite migrations occurred at these sites.

## Discussion

In this study, homology modeling and *in silico* protein glycosylation of representative HA and NA proteins as well as amino acid variability analysis at antigenic sites were employed for predicting biological functions of glycosite migrations in the host adaptation of human seasonal influenza H1N1 viruses. After modeling the structures of representative HA and NA proteins (including their different patterns of potential N-glycosites) from human influenza A (H1N1) viruses, complex glycans lacking terminal sialic acid residues were added *in silico* onto each variable glycosite using the Glyprot server. The structure of the glycans attached to each site may differ since glycan structure on HA and NA is mainly determined by the host and by the location of the glycosite for influenza viruses. Mammalian cells possess a great deal of glycosylating enzymes and thus generally add large, tri- and tetra-antennary oligosaccharides onto the HA and NA of influenza viruses [8], [36]. Here, these glycans only consisted of nine monosaccharides without terminal sialic acid residues were used in this study, which should be suitable to model the natural state of the viral glycans as far as possible for a fair prediction of the possible function of the glycans attached at various important glycosites.

In influenza H1N1 viruses, some of the potential glycosites, such as glycosites 28, 40, 104, 304, 498 and 557 on HA and glycosites 58, 63, 88, 146 and 235 on NA, were highly conserved in all strains isolated from various animals and humans and therefore appeared to be essential for the formation and/or maintenance of functional HA and NA. While some other glycosites, such as glycosites 142, 144, 172, 177 and 179 on the top of the HA head, glycosites 71 and 286 on the side of the HA head and glycosites 365, 434 and 455 around the enzymatic active centre of NA, only appeared during certain evolutionary periods for the human seasonal influenza H1N1 viruses [25]. Glycosylation at any one of these sites is neither prohibited nor required for the formation of the functional HA, glycan diversity might have a major selective effect for function of the HA. The presence or absence, the location and the structures of the glycans for those variable sites could determine how well the virus grows in certain species of cells.

It has been proven that the acquisition of potential glycosylation sites is one of the effective ways for influenza viruses to escape positive selective pressures from the hosts [7], [8]. Here, several possible biological functions of glycosite migration for the host adaptation of human seasonal influenza H1N1 viruses were summarized as below based on the analysis above:

The first possible biological function of glycosite migrations is to more effectively mask the antigenic sites of HA and NA, which is very important for viral immune evasion from host. Some new glycosites were added to mask antigenic sites, while some positional conversions of the glycosites were mainly to increase the effectiveness with which the glycan masks the antigenic sites. For example, the glycans attached to glycosites 144 and 179 of HA could mask the HA antigenic site Sa, however, the glycans attached to glycosites 172, 142 and 177 of HA might be more effective to mask it.

The second possible biological function is to more effectively protect the enzymatic cleavage sites of NA. One possible function of the glycans attached to the glycosites on the stalk of NA and to glycosite 235 is to protect the enzymatic cleavage sites on the stalk. The positional conversion of the glycosites on the stalk of NA (such as the conversion from residues 50 to 44 and from 68 to 70) could more effectively protect the enzymatic cleavage sites on the stalk of NA.

The third possible biological function is to stabilize the structures of the polymeric glycoproteins. The addition or positional conversion of glycosites to the subunit interfaces of HA and NA may function to stabilize the trimeric structures of HA and NA, respectively. This could be the case for the glycans attached to glycosites 179, 177, 71 and 104 of HA and to glycosites 146, 434 and 455 of NA. For example, twelve glycans attached at glycosites 146, 434 and 455 on each tetrameric NA (1986–2009 strains) could cover almost all of the interfaces between subunits of the NA, which should efficiently enhance the stability of the NA (Figure 5a).

The fourth possible biological function is to regulate the activity of HA and NA. The positional conversion of glycosite 179 to glycosite 177 on HA might effectively reduce the obstruction of the receptor binding sites on the neighboring subunit and thus increase the receptor binding activity of HA. The positional conversion of glycosite 104 to glycosite 71 on HA could also effectively reduce the obstruction of the receptor binding site. Glycosites 146, 365 and 434 were located around the enzymatic active site, and therefore the glycans attached to these sites may regulate the catalytic activity of NA.

The fifth possible biological function is to balance the binding activity of HA with the releasing activity of NA. In fact, HA and NA together determine the traits of the influenza viruses, such as the host range, virulence and infectivity. It is necessary for the influenza virus to balance the host receptor binding activity of HA with the releasing activity of NA in the viral life cycle, and glycosite number was one of the important mediation factors by mediating their proper steric structures, influencing their activity and promoting the infection and spread of the influenza virus. [37], [38], [39]. If the binding strength between the host receptor and HA increases when HA has fewer carbohydrate modifications, then the activity of NA must also increase in order to promote the release of the viral particle from the surface of the host cell. Conversely, when HA is extensively glycosylated, it may interact weakly with the host receptors. At this time, the influenza virus would require a less active NA to facilitate the release of the viral particle. Moreover, it has been reported that the HA and NA of the pandemic 1918 and 2009 influenza viruses need to be correctly paired (HA 1918+NA 1918, HA 2009+NA 2009) to achieve the highest infectious activity [40]. The glycosite migration should have the same functions in the activity mediation of HA and NA as glycosite numbers. The nearly identical evolutionary process and phases of glycosites on both HA and NA proteins described previously (glycosite addition or migration occurred on both HA and NA almost synchronously) could account for the requirement of corresponding matching patterns of glycosylation on the HA and NA of influenza viruses (Figure S1) [25].

Besides, glycosite migrations may also play an important role in coordinating the function of the glycans at different glycosites. When one glycan can shield an antigenic site or enzymatic cleavage site effectively after it transfers from one glycosite to another, some of the other glycans may also need to transfer for protecting other regions. This may happen between the positional conversions of glycosite 179 to 177 and glycosite 144 to 172 and then to 142. When antigenic Sb site of HA can be protected well by glycans at glycosite 177 on the adjacent subunit, the glycans at glycosite 144 may need to transfer to site 172 and then to site 142 to shield antigenic Sa site more effectively.

Since the addition of glycans around the receptor binding site of HA and the enzymatic active centre of NA can have either positive or detrimental effects on the virus–while it shields antigenic sites against immune recognition, it reduces receptor affinity of HA and enzymatic activity of NA [8], [15], glycosite migration (with no glycan added) may be one of the artful manners for human seasonal influenza viruses to maximize the ratio of positive to detrimental effects of each added glycan.

## Supporting Information

Figure S1 **Coordination of glycosite alterations between HA and NA of human seasonal influenza H1N1 viruses** [**25**] **.** (A) The alteration process of glycosites on the head of HA. (B) The alteration process of glycosites on the side of HA. (C) The alteration process of glycosites on the head of NA. (D) The alteration process of glycosites on the stalk of NA. The dotted lines represented the superficial alterations based on genome-based analysis, while the corresponding full lines illustrated the possibly alteration processes after further analysis by homology modeling and *in silico* protein glycosylation.
(TIF)

Table S1 **Cross-neutralization among vaccine strains.** Hemagglutination inhibition titers. (Homologous titers are marked in bold. > = <40) [31].
(DOC)

Table S2 **The potential glycosites of HA in vaccine strains since 1977.** The sequence in each section of the table represents the corresponding sequon of each site. Potential glycosites are highlighted in yellow.
(DOC)

Table S3 **The potential glycosites of NA in vaccine strains since 1977.** The sequence in each section of the table represents the corresponding sequon of each site. Potential glycosites are highlighted in yellow.
(DOC)

Table S4 **The tryptic cleavage sites and potential glycosites on the NA stalk of human influenza viruses (both pandemic and seasonal).** The conservation of tryptic cleavage sites and potential glycosites were shown as percentage (‘%’ had been omitted) and were highlighted in olive green and orange, respectively. The numbers of corresponding strains used for the analysis were given in the brackets.
(DOC)
