# Supplementary figures and images for: Prediction of Biological Functions on Glycosylation Site Migrations in Human Influenza H1N1 Viruses
Source: PLoS One. 2012 Feb 15;7(2):e32119. doi: 10.1371/journal.pone.0032119 (PMC3280219; doi:10.1371/journal.pone.0032119)

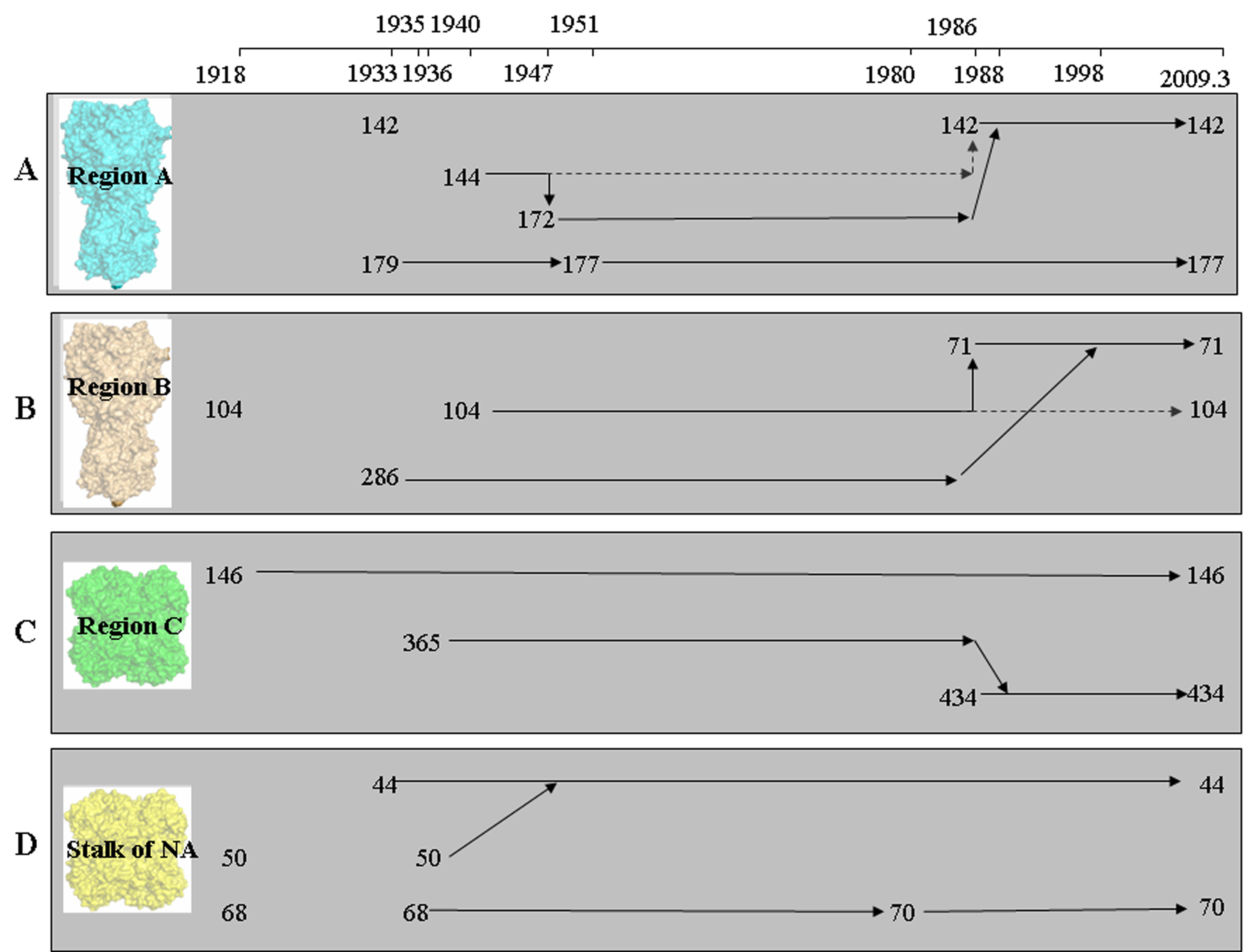

Supplement: Figure S1 — Coordination of glycosite alterations between HA and NA of human seasonal influenza H1N1 viruses [25] . (A) The alteration process of glycosites on the head of HA. (B) The alteration process of glycosites on the side of HA. (C) The alteration process of glycosites on the head of NA. (D) The alteration process of glycosites on the stalk of NA. The dotted lines represented the superficial alterations based on genome-based analysis, while the corresponding full lines illustrated the possibly alteration processes after further analysis by homology modeling and in silico protein glycosylation. (TIF) [file pone.0032119.s001.tif]
