# Supplementary material for: Prediction of Biological Functions on Glycosylation Site Migrations in Human Influenza H1N1 Viruses
Source: PLoS One. 2012 Feb 15;7(2):e32119. doi: 10.1371/journal.pone.0032119 (PMC3280219; doi:10.1371/journal.pone.0032119)
Supplement: Table S1 — Cross-neutralization among vaccine strains. Hemagglutination inhibition titers. (Homologous titers are marked in bold. > = <40) [31]. (DOC) [file pone.0032119.s002.doc]

Table S1. Cross-neutralization among vaccine strains: Hemagglutination inhibition titers. (Homologous titers are marked in bold. ＞ = ＜40) .

|  | post-infection ferret sera | | | | | | | | Group |
| --- | --- | --- | --- | --- | --- | --- | --- | --- | --- |
| Virus | A/USSR90/77 | A/Brazil  11/78 | A/Chile  1/83 | A/Sing  6/86 | A/Taiw  01/86 | A/Tex  36/91 | A/Beij  262/95 | A/NC  20/99 |
| A/USSR/90/77 | **1280** | 640 | 40 | ﹤ | ﹤ | ﹤ | ﹤ | ﹤ | Group Ⅰ |
| A/Brazil/11/78 | 320 | **1280** | 80 | ﹤ | ﹤ | ﹤ | ﹤ | ﹤ |
| A/Chile/1/83 | 80 | 80 | **320** | ﹤ | ﹤ | ﹤ | ﹤ | ﹤ |
| A/Singapore/6/86 | ﹤ | ﹤ | ﹤ | **1280** | 320 | 1280 | 1280 | ﹤ | Group Ⅱ |
| A/Taiwan/01/86 | ﹤ | ﹤ | ﹤ | 640 | **640** | 1280 | 1280 | ﹤ |
| A/Texas/36/91 | ﹤ | ﹤ | ﹤ | 1280 | 640 | **2560** | 1280 | 40 |
| A/Beijing/262/95 | ﹤ | ﹤ | ﹤ | 640 | 640 | 2560 | **2560** | 40 |
| A/New Caledonia/20/99 | ﹤ | ﹤ | ﹤ | ﹤ | ﹤ | ﹤ | ﹤ | **640** | Group Ⅲ |
