# Supplementary material for: Prediction of Biological Functions on Glycosylation Site Migrations in Human Influenza H1N1 Viruses
Source: PLoS One. 2012 Feb 15;7(2):e32119. doi: 10.1371/journal.pone.0032119 (PMC3280219; doi:10.1371/journal.pone.0032119)
Supplement: Table S2 — The potential glycosites of HA in vaccine strains since 1977. The sequence in each section of the table represents the corresponding sequon of each site. Potential glycosites are highlighted in yellow. (DOC) [file pone.0032119.s003.doc]

Table S2. The potential glycosites of HA in vaccine strains since 1977. The sequence in each section of the table represents the corresponding sequon of each site. Potential glycosites are highlighted in yellow.

| Virus | 28 | 40 | 71 | 104 | 142 | 144 | 172 | 177 | 286 | 304 | 498 | 557 | Group |
| --- | --- | --- | --- | --- | --- | --- | --- | --- | --- | --- | --- | --- | --- |
| A/USSR/90/77 | **NST** | **NVT** | KCN | **NGT** | KHN | **NVT** | **NGS** | **NLS** | **NAS** | **NSS** | **NGT** | **NGS** | Group Ⅰ |
| A/Brazil/11/78 | **NST** | **NVT** | KCS | **NGT** | KHN | **NIT** | **NGS** | **NLS** | **NAS** | **NSS** | **NGT** | **NGS** |
| A/Chile/1/83 | **NST** | **NVT** | KCS | **NGT** | KHN | **NVT** | **NGS** | **NLS** | **NAS** | **NSS** | **NGT** | **NGS** |
| A/Singapore/6/86 | **NST** | **NVT** | **NCS** | **NGT** | **NHT** | TVT | **NGS** | **NLS** | **NAS** | **NSS** | **NGT** | **NGS** | Group Ⅱ |
| A/Taiwan/01/86 | **NST** | **NVT** | **NCS** | **NGT** | **NHT** | TVT | **NGS** | TLS | **NAS** | **NSS** | **NGT** | **NGS** |
| A/Texas/36/91 | **NST** | **NVT** | **NCS** | **NGT** | **NHT** | TVT | NGL | **NLS** | **NAS** | **NSS** | **NGT** | **NGS** |
| A/Beijing/262/95 | **NST** | **NVT** | **NCS** | **NGT** | KHT | TVT | NGL | **NLS** | NAP | **NSS** | **NGT** | **NGS** |
| A/New Caledonia/20/99 | **NST** | **NVT** | **NCS** | **NGT** | **NHT** | TVT | NGL | **NLS** | NAP | **NSS** | **NGT** | **NGS** | Group Ⅲ |
