# Supplementary material for: Prediction of Biological Functions on Glycosylation Site Migrations in Human Influenza H1N1 Viruses
Source: PLoS One. 2012 Feb 15;7(2):e32119. doi: 10.1371/journal.pone.0032119 (PMC3280219; doi:10.1371/journal.pone.0032119)
Supplement: Table S4 — The tryptic cleavage sites and potential glycosites on the NA stalk of human influenza viruses (both pandemic and seasonal). The conservation of tryptic cleavage sites and potential glycosites were shown as percentage (‘%’ had been omitted) and were highlighted in olive green and orange, respectively. The numbers of corresponding strains used for the analysis were given in the brackets. (DOC) [file pone.0032119.s005.doc]

**Table S4. The tryptic cleavage sites and potential glycosites on the NA stalk of human influenza viruses (both pandemic and seasonal).** The conservation of tryptic cleavage sites and potential glycosites were shown as percentage (‘%’ had been omitted) and were highlighted in olive green and orange, respectively. The numbers of corresponding strains used for the analysis were given in the brackets.

|  | **44** | **50** | **52** | **57** | **58** | **63** | **68** | **70** | **78** | **80** | **84** | **88** |
| --- | --- | --- | --- | --- | --- | --- | --- | --- | --- | --- | --- | --- |
| 1933 (9) | 100 |  |  | 66.67 |  |  |  |  |  |  |  | 100 |
| 1934 (7) | 100 |  |  | 100 | 100 |  |  |  | 100 |  |  | 100 |
| 1935-1947 (19) | 89.47 | 73.68 |  | 78.95 | 89.47 | 94.74 | 89.47 |  | 78.95 |  |  | 100 |
| 1948-1979 (36) | 100 |  | 100 |  | 100 | 100 | 100 |  | 100 |  |  | 100 |
| 1980-1985 (60) | 100 |  | 100 |  | 100 | 100 | 1.67 | 98.33 | 100 |  |  | 100 |
| 1986-1987 (10) | 100 |  | 100 |  | 100 | 100 |  | 100 |  |  |  | 100 |
| 1988-2009 (1891) | 99.89 | 0.05 | 100 |  | 99.95 | 100 |  | 99.84 | 53.94 | 99.68 |  | 99.79 |
|  |  |  |  |  |  |  |  |  |  |  |  |  |
| Human pandemic 1918 (1) |  | 100 |  |  | 100 | 100 | 100 |  |  |  |  | 100 |
| Human pandemic 2009 (702) |  | 100 |  |  | 100 | 100 | 100 |  |  |  | 100 | 99.86 |
